# Crystal structure of an Hg^II^ coordination polymer with an unsymmetrical dipyridyl ligand: *catena*-poly[[[di­chlorido­mercury(II)]-μ-*N*-(pyridin-4-ylmeth­yl)pyridin-3-amine-κ^2^ *N*:*N*′] chloro­form hemisolvate]

**DOI:** 10.1107/S2056989016015310

**Published:** 2016-10-04

**Authors:** Suk-Hee Moon, Donghyun Kang, Ki-Min Park

**Affiliations:** a Department of Food and Nutrition, Kyungnam College of Information and Technology, Busan 47011, Republic of Korea; b Department of Science Education, Kyungnam University, Changwon 51767, Republic of Korea; c Research Institute of Natural Science, Gyeongsang National University, Jinju 52828, Republic of Korea

**Keywords:** crystal structure, Hg^II^ compound, unsymmetrical dipyridyl ligand, zigzag coordination polymer

## Abstract

The asymmetric unit of the title compound, {[Hg*L*Cl_2_]·0.5CHCl_3_}_*n*_ (*L* = *N*-(pyridin-4-ylmeth­yl)pyridin-3-amine, C_11_H_11_N_3_), contains one Hg^II^ ion, one bridging *L* ligand, two chloride ligands and a chloro­form solvent mol­ecule with half-occupancy that is disordered about a crystallographic twofold rotation axis. Each Hg^II^ ion is coordinated by two pyridine N atoms from two symmetry-related *L* ligands and two chloride anions in a highly distorted tetra­hedral geometry with bond angles falling in the range 99.05 (17)–142.96 (7)°. Each *L* ligand bridges two Hg^II^ ions, forming polymeric zigzag chains propagating in [010]. In the crystal, the chains are linked by inter­molecular N/C—H⋯Cl hydrogen bonds together with weak C—H⋯π inter­actions, resulting in the formation of a three-dimensional supra­molecular network, which is further stabilized by C—Cl⋯π inter­actions between the solvent chloro­form mol­ecules and the pyridine rings of *L* [chloride-to-centroid distances = 3.442 (11) and 3.626 (13) Å]. In addition, weak Cl⋯Cl contacts [3.320 (5) Å] between the chloro­form solvent mol­ecules and the coordinating chloride anions are also observed.

## Abstract 2

In the title compound, each Hg^II^ ion is coordinated by two pyridine N atoms from two symmetry-related unsymmetrical dipyridyl ligands and two chloride anions in a highly distorted tetra­hedral geometry. Each unsymmetrical dipyridyl ligand links two Hg^II^ ions into polymeric zigzag chains. In the crystal, the chains are linked into a three-dimensional supra­molecular network by inter­molecular N/C—H⋯Cl hydrogen bonds and weak C—H⋯π inter­actions. Weak C—Cl⋯π inter­actions and Cl⋯Cl contacts between the network and the solvent chloro­form mol­ecules are also observed.

## Chemical context   

A variety of coordination polymers have been explored extensively over the last two decades because of their fascinating architectures and their useful applications in materials chemistry (Silva *et al.*, 2015 ▸; Furukawa *et al.*, 2014 ▸; Robson, 2008 ▸; Leong & Vittal, 2011 ▸). In this area of research, symmetrical dipyridyl ligands composed of two terminal pyridines with same substituted nitro­gen positions have been used mainly for the design and construction of the coordination polymers. By contrast, investigations based on unsymmetrical dipyridyl ligands, with the nitro­gen atoms in different positions on each of the two terminal pyridines, are still rare (Uemura *et al.*, 2008 ▸; Khlobystov *et al.*, 2003 ▸). Recently, our group and that of Gao have already reported Ag^I^ coordination polymers with some unsymmetrical dipyridyl ligands such as *N*-(pyridine-3-ylmeth­yl)pyridine-2-amine (Lee *et al.*, 2013 ▸; Zhang *et al.*, 2013 ▸), *N*-(pyridine-2-ylmeth­yl)pyridine-3-amine (Ju *et al.*, 2014 ▸; Moon & Park, 2014 ▸; Moon *et al.*, 2014 ▸; Zhang *et al.*, 2013 ▸) and *N*-(pyridine-4-ylmeth­yl)pyridine-3-amine (Lee *et al.*, 2015 ▸; Moon *et al.*, 2015 ▸; Zhang *et al.*, 2013 ▸). As a part of our ongoing efforts to construct coordination polymers with such unsymmetrical dipyridyl ligands, we prepared the title compound obtained by the reaction of mercury(II) chloride with an unsymmetrical dipyridyl ligand, namely *N*-(pyridine-4-ylmeth­yl)pyridine-3-amine, synthesized according to a literature procedure (Lee *et al.*, 2013 ▸). Herein, we report the crystal structure of the title compound.
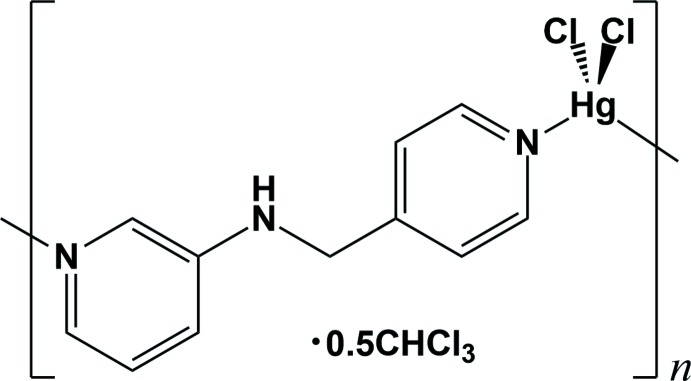


## Structural commentary   

**Figure 1:**
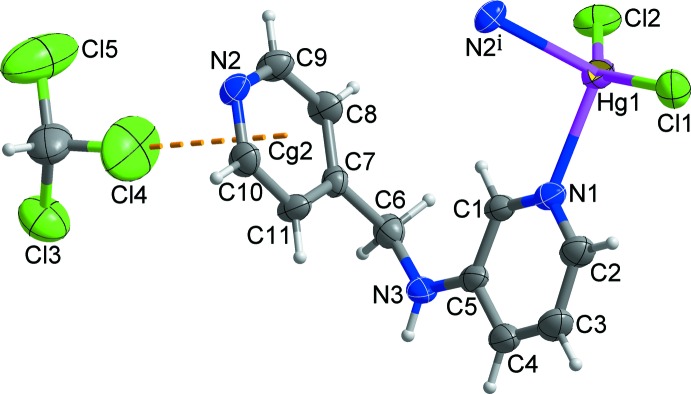
A view of the mol­ecular structure of the title compound, showing the atom-numbering scheme [symmetry code: (i) −*x* + 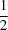, *y* + 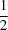, −*z* + 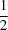]. Displacement ellipsoids are drawn at the 30% probability level. Only one component of the disordered chloro­form mol­ecule is shown. The dashed line represents the inter­molecular C—Cl⋯π inter­action [Cl4⋯*Cg*2 = 3.442 (11) Å; *Cg*2 is the centroid of the N2/C7–C11 ring].

The asymmetric unit of the title compound, {[Hg*L*Cl_2_]·0.5CHCl_3_}_*n*_, *L* = *N*-(pyridine-4-ylmeth­yl)pyridine-3-amine, C_11_H_11_N_3_, comprises one Hg^II^ ion, one *L* ligand, two chloride anions and one half-mol­ecule of chloro­form. The solvent mol­ecule is disordered over two orientations of equal occupancy about the crystallographic twofold rotation axis. As shown in Fig. 1 ▸, the coordination geometry of each Hg^II^ ion is highly distorted tetra­hedral with two coordination sites being occupied by two pyridine N atoms from two symmetry-related *L* ligands. The geometry of the Hg^II^ ion is completed by the coordination of two chloride ions. The tetra­hedral angles around the Hg^II^ ion fall in the range of 99.05 (17)–142.96 (7)° (Table 1 ▸).

**Figure 2:**
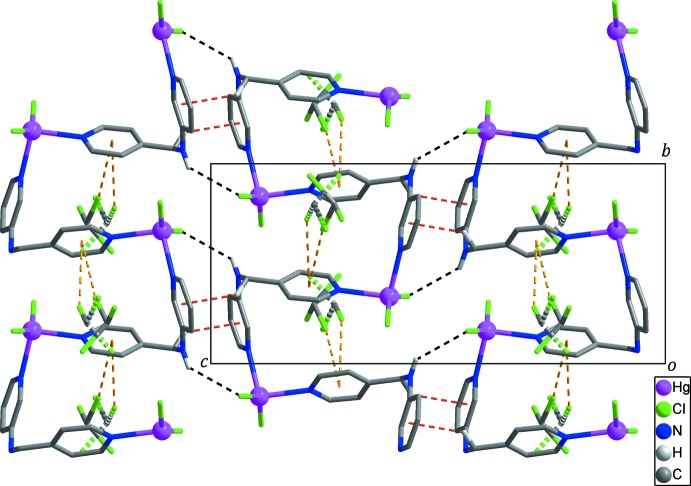
The layer formed through inter­molecular N—H⋯Cl hydrogen bonds (black dashed lines) and weak C—H⋯π inter­actions (red dashed lines). Disordered chloro­form mol­ecules and inter­molecular C—Cl⋯π inter­actions are shown as two-colored dashed lines and yellow dashed lines, respectively. H atoms not involved in inter­molecular inter­actions have been omitted for clarity.

Each *L* ligand bridges two Hg^II^ ions into an infinite zigzag chain propagating along the *b* axis (Fig. 2 ▸). The separation between the Hg^II^ ions through a *L* ligand in the chain is 8.1033 (6) Å. In the *L* ligand, the C_py_—N—C—C_py_ torsion angle is −70.9 (7)° while the dihedral angle between two terminal pyridine ring planes is 85.0 (2)°. The conformation of the *L* ligand, along with the the N_py_—Hg—N_py_ coordination angle [99.05 (17)°], may induce the zigzag topology of the chain.

## Supra­molecular features   

**Figure 3:**
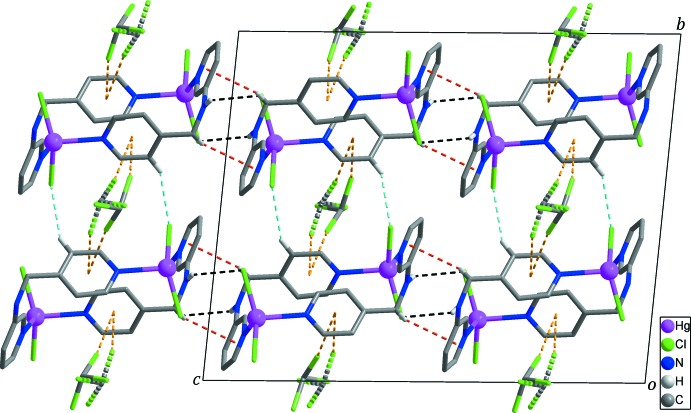
The three-dimensional supra­molecular network constructed through inter­molecular C—H⋯Cl hydrogen bonds (light-blue dashed lines) and C—Cl⋯π inter­actions (yellow dashed lines) between the layers formed through N—H⋯Cl (black dashed lines) and C—H⋯π (red dashed lines) inter­actions. Disordered chloro­form mol­ecules are shown as two-colored dashed lines. H atoms not involved in inter­molecular inter­actions have been omitted for clarity.

In the crystal, adjacent zigzag chains are linked by inter­molecular N—H⋯Cl hydrogen bonds and weak inter­molecular C—H⋯π inter­actions (Table 2 ▸), forming a layer extending parallel to the *bc* plane (Figs. 2 ▸ and 3 ▸). Furthermore, neighboring layers are packed by C—H⋯Cl hydrogen bonds (Table 2 ▸), resulting in the formation of a three-dimensional supra­molecular network (Fig. 3 ▸). This three-dimensional network is further stabilized by C—Cl⋯π inter­actions (Chifotides & Dunbar, 2013 ▸; Matter *et al.*, 2009 ▸) between the solvent chloro­form mol­ecules and the pyridine rings of *L* with Cl4⋯*Cg*2 = 3.442 (11) Å, C12—Cl4⋯*Cg*2 = 170.7 (8)°, Cl5⋯*Cg*2^iv^ = 3.626 (13) Å and C12—Cl5⋯*Cg*2^iv^ 144.1 (8)° [yellow dashed lines in Figs. 1 ▸, 2 ▸ and 3 ▸; *Cg*2 is the centroid of the N2/C7–C11 ring; symmetry code: (iv) −*x*, *y*, −*z* + 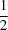]. In addition, weak inter­molecular Cl⋯Cl contacts between the solvent chloro­form mol­ecule and the coordinating chloride anion [Cl1⋯Cl3^v^ = 3.320 (5) Å, Hg1—Cl1⋯Cl3^v^ = 126.70 (14) and Cl1⋯Cl3^v^—C12^v^ = 169.2 (8)°; symmetry code: (v) *x* + 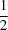, *y* + 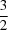, *z*] are observed.

## Synthesis and crystallization   

The *L* ligand was synthesized according to a literature method (Lee *et al.*, 2013 ▸). X-ray-quality single crystals of the title compound were obtained by slow diffusion of a methanol solution of HgCl_2_ into a chloro­form solution of the *L* ligand.

## Refinement   

Crystal data, data collection and structure refinement details are summarized in Table 3 ▸. A reflection affected by the beamstop was omitted from the final refinement. The chloro­form mol­ecule is disordered over two sets of sites about a twofold rotation axis with equal occupancy. The C—Cl bond lengths were restrained using the DFIX instructions in *SHELXL2014/7* (Sheldrick, 2015 ▸). All H atoms were positioned geometrically with *d*(C—H) = 0.93 Å for C*sp* ^2^—H, 0.97 Å for methyl­ene C—H, 0.98 Å for methine C—H, and 0.86 Å for amine N—H, and were refined as riding with *U*_iso_(H) = 1.2*U*_eq_(C,N).

## Supplementary Material

Crystal structure: contains datablock(s) I, New_Global_Publ_Block. DOI: 10.1107/S2056989016015310/sj5509sup1.cif

Structure factors: contains datablock(s) I. DOI: 10.1107/S2056989016015310/sj5509Isup2.hkl

CCDC reference: 1507232

Additional supporting information: crystallographic information; 3D view; checkCIF report

## Figures and Tables

**Table 1:** Selected geometric parameters (Å, °)

Hg1—N1	2.367 (5)	Hg1—Cl1	2.3759 (18)
Hg1—Cl2	2.3718 (19)	Hg1—N2^i^	2.385 (5)
			
N1—Hg1—Cl2	103.82 (14)	N1—Hg1—N2^i^	99.05 (17)
N1—Hg1—Cl1	102.31 (14)	Cl2—Hg1—N2^i^	101.20 (14)
Cl2—Hg1—Cl1	142.96 (7)	Cl1—Hg1—N2^i^	100.11 (13)

Symmetry code: (i) 
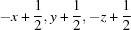
.

**Table 2:** Hydrogen-bond geometry (Å, °) *Cg*1 is the centroid of the N1/C1–C5 ring.

*D*—H⋯*A*	*D*—H	H⋯*A*	*D*⋯*A*	*D*—H⋯*A*
N3—H3⋯Cl2^ii^	0.86	2.78	3.467 (5)	138
C8—H8⋯Cl1^iii^	0.93	2.80	3.654 (6)	153
C6—H6*B*⋯*Cg*1^ii^	0.97	2.71	3.465 (7)	135

Symmetry codes: (ii) 
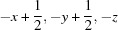
; (iii) 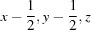.

**Table 3:** Experimental details

Crystal data	
Chemical formula	[HgCl_2_(C_11_H_11_N_3_)]·0.5CHCl_3_
*M*_r_	516.40
Crystal system, space group	Monoclinic, *C*2/*c*
Temperature (K)	298
*a*, *b*, *c* (Å)	16.6906 (14), 9.1942 (8), 21.0159 (17)
β (°)	95.501 (2)
*V* (Å^3^)	3210.2 (5)
*Z*	8
Radiation type	Mo *K*α
μ (mm^−1^)	10.16
Crystal size (mm)	0.4 × 0.3 × 0.3

Data collection	
Diffractometer	Bruker APEXII CCD area detector
Absorption correction	Multi-scan (*SADABS*; Bruker, 2014 ▸)
*T*_min_, *T*_max_	0.521, 0.928
No. of measured, independent and observed [*I* > 2σ(*I*)] reflections	8852, 3151, 2189
*R*_int_	0.044
(sin θ/λ)_max_ (Å^−1^)	0.617

Refinement	
*R*[*F* ^2^ > 2σ(*F* ^2^)], *wR*(*F* ^2^), *S*	0.033, 0.074, 0.99
No. of reflections	3151
No. of parameters	190
No. of restraints	3
H-atom treatment	H-atom parameters constrained
Δρ_max_, Δρ_min_ (e Å^−3^)	0.58, −0.83

Computer programs: *APEX2* and *SAINT* (Bruker, 2014 ▸), *SHELXS97* and *SHELXTL* (Sheldrick, 2008 ▸), *SHELXL2014* (Sheldrick, 2015 ▸) and *DIAMOND* (Brandenburg, 2010 ▸).

## supplementary crystallographic information

### Crystal data

**Table:**

[HgCl_2_(C_11_H_11_N_3_)]·0.5CHCl_3_	*F*(000) = 1928
*M**_r_* = 516.40	*D*_x_ = 2.137 Mg m^−^^3^
Monoclinic, *C*2/*c*	Mo *K*α radiation, λ = 0.71073 Å
*a* = 16.6906 (14) Å	Cell parameters from 3870 reflections
*b* = 9.1942 (8) Å	θ = 2.0–28.3°
*c* = 21.0159 (17) Å	µ = 10.16 mm^−^^1^
β = 95.501 (2)°	*T* = 298 K
*V* = 3210.2 (5) Å^3^	Block, colorless
*Z* = 8	0.4 × 0.3 × 0.3 mm

### Data collection

**Table:**

Bruker APEXII CCD area detector diffractometer	2189 reflections with *I* > 2σ(*I*)
phi and ω scans	*R*_int_ = 0.044
Absorption correction: multi-scan (SADABS; Bruker, 2014)	θ_max_ = 26.0°, θ_min_ = 2.5°
*T*_min_ = 0.521, *T*_max_ = 0.928	*h* = −12→20
8852 measured reflections	*k* = −11→10
3151 independent reflections	*l* = −24→25

### Refinement

**Table:**

Refinement on *F*^2^	3 restraints
Least-squares matrix: full	Hydrogen site location: inferred from neighbouring sites
*R*[*F*^2^ > 2σ(*F*^2^)] = 0.033	H-atom parameters constrained
*wR*(*F*^2^) = 0.074	*w* = 1/[σ^2^(*F*_o_^2^) + (0.034*P*)^2^] where *P* = (*F*_o_^2^ + 2*F*_c_^2^)/3
*S* = 0.99	(Δ/σ)_max_ = 0.001
3151 reflections	Δρ_max_ = 0.58 e Å^−^^3^
190 parameters	Δρ_min_ = −0.83 e Å^−^^3^

### Special details

**Table:**

Geometry. All esds (except the esd in the dihedral angle between two l.s. planes) are estimated using the full covariance matrix. The cell esds are taken into account individually in the estimation of esds in distances, angles and torsion angles; correlations between esds in cell parameters are only used when they are defined by crystal symmetry. An approximate (isotropic) treatment of cell esds is used for estimating esds involving l.s. planes.

### Fractional atomic coordinates and isotropic or equivalent isotropic displacement parameters (Å^2^)

**Table:**

	*x*	*y*	*z*	*U*_iso_*/*U*_eq_	Occ. (<1)
Hg1	0.32126 (2)	0.66479 (3)	0.10699 (2)	0.06180 (11)	
Cl1	0.44104 (11)	0.8055 (2)	0.11123 (9)	0.0766 (5)	
Cl2	0.18389 (11)	0.6629 (2)	0.06581 (9)	0.0839 (6)	
N1	0.3676 (3)	0.4300 (6)	0.0825 (2)	0.0548 (13)	
N2	0.1898 (3)	0.1323 (5)	0.2816 (2)	0.0568 (13)	
N3	0.2853 (4)	0.0648 (6)	0.0611 (2)	0.0641 (15)	
H3	0.3015	−0.0182	0.0486	0.077*	
C1	0.3176 (4)	0.3179 (7)	0.0811 (3)	0.0522 (15)	
H1	0.2663	0.3330	0.0938	0.063*	
C2	0.4398 (4)	0.4134 (8)	0.0648 (3)	0.0693 (18)	
H2	0.4749	0.4922	0.0662	0.083*	
C3	0.4649 (5)	0.2790 (10)	0.0439 (4)	0.080 (2)	
H3A	0.5163	0.2687	0.0310	0.096*	
C4	0.4152 (5)	0.1641 (8)	0.0423 (3)	0.075 (2)	
H4	0.4322	0.0742	0.0284	0.090*	
C5	0.3372 (4)	0.1798 (7)	0.0618 (3)	0.0562 (16)	
C6	0.2052 (4)	0.0749 (7)	0.0800 (3)	0.0615 (17)	
H6A	0.1761	−0.0130	0.0667	0.074*	
H6B	0.1780	0.1559	0.0576	0.074*	
C7	0.2007 (4)	0.0955 (7)	0.1513 (2)	0.0499 (15)	
C8	0.1386 (4)	0.1686 (7)	0.1738 (3)	0.0636 (17)	
H8	0.0986	0.2093	0.1454	0.076*	
C9	0.1348 (4)	0.1821 (7)	0.2375 (3)	0.0664 (18)	
H9	0.0904	0.2298	0.2512	0.080*	
C10	0.2502 (4)	0.0629 (7)	0.2600 (3)	0.0677 (19)	
H10	0.2895	0.0249	0.2897	0.081*	
C11	0.2594 (4)	0.0424 (7)	0.1953 (3)	0.0618 (18)	
H11	0.3040	−0.0060	0.1825	0.074*	
C12	0.0095 (13)	−0.2965 (16)	0.2726 (7)	0.128 (8)	0.5
H12	0.0355	−0.3356	0.3128	0.153*	0.5
Cl3	−0.0016 (4)	−0.4392 (6)	0.2177 (3)	0.1288 (19)	0.5
Cl4	0.0705 (7)	−0.1706 (11)	0.2464 (6)	0.201 (5)	0.5
Cl5	−0.0803 (8)	−0.2208 (14)	0.2885 (6)	0.230 (7)	0.5

### Atomic displacement parameters (Å^2^)

**Table:**

	*U*^11^	*U*^22^	*U*^33^	*U*^12^	*U*^13^	*U*^23^
Hg1	0.06149 (17)	0.07271 (18)	0.05193 (16)	0.00720 (15)	0.00918 (12)	−0.00275 (14)
Cl1	0.0576 (10)	0.0833 (12)	0.0887 (13)	0.0035 (9)	0.0056 (10)	0.0198 (10)
Cl2	0.0639 (11)	0.1104 (15)	0.0748 (11)	0.0191 (11)	−0.0065 (9)	−0.0262 (11)
N1	0.052 (3)	0.069 (3)	0.044 (3)	0.012 (3)	0.006 (3)	−0.001 (2)
N2	0.053 (3)	0.078 (4)	0.040 (3)	0.007 (3)	0.009 (3)	0.006 (2)
N3	0.096 (4)	0.052 (3)	0.047 (3)	0.018 (3)	0.019 (3)	−0.004 (2)
C1	0.053 (4)	0.063 (4)	0.041 (3)	0.017 (3)	0.007 (3)	−0.001 (3)
C2	0.056 (4)	0.083 (5)	0.070 (4)	0.019 (4)	0.011 (4)	0.008 (4)
C3	0.065 (5)	0.097 (6)	0.083 (6)	0.030 (5)	0.026 (4)	0.020 (5)
C4	0.099 (6)	0.072 (5)	0.057 (4)	0.032 (5)	0.027 (4)	0.010 (4)
C5	0.070 (4)	0.066 (4)	0.034 (3)	0.023 (4)	0.010 (3)	0.007 (3)
C6	0.074 (5)	0.061 (4)	0.047 (4)	0.002 (4)	−0.004 (4)	0.001 (3)
C7	0.061 (4)	0.053 (3)	0.036 (3)	0.000 (3)	0.002 (3)	0.006 (3)
C8	0.052 (4)	0.082 (4)	0.053 (4)	0.017 (4)	−0.011 (3)	0.007 (4)
C9	0.052 (4)	0.089 (5)	0.059 (4)	0.015 (4)	0.005 (3)	−0.002 (4)
C10	0.065 (4)	0.083 (5)	0.055 (4)	0.018 (4)	0.004 (4)	0.017 (3)
C11	0.061 (4)	0.083 (5)	0.042 (3)	0.029 (4)	0.008 (3)	0.003 (3)
C12	0.18 (2)	0.108 (14)	0.098 (18)	0.000 (18)	0.04 (2)	−0.024 (10)
Cl3	0.147 (5)	0.100 (3)	0.135 (4)	−0.006 (4)	−0.011 (5)	−0.026 (3)
Cl4	0.208 (10)	0.167 (7)	0.234 (11)	−0.120 (7)	0.051 (8)	−0.057 (7)
Cl5	0.184 (9)	0.242 (12)	0.281 (15)	−0.077 (9)	0.110 (10)	−0.150 (11)

### Geometric parameters (Å, º)

**Table:**

Hg1—N1	2.367 (5)	C4—C5	1.409 (10)
Hg1—Cl2	2.3718 (19)	C4—H4	0.9300
Hg1—Cl1	2.3759 (18)	C6—C7	1.519 (7)
Hg1—N2^i^	2.385 (5)	C6—H6A	0.9700
N1—C2	1.303 (8)	C6—H6B	0.9700
N1—C1	1.326 (8)	C7—C8	1.358 (8)
N2—C10	1.310 (8)	C7—C11	1.371 (8)
N2—C9	1.323 (8)	C8—C9	1.352 (9)
N2—Hg1^ii^	2.386 (5)	C8—H8	0.9300
N3—C5	1.366 (8)	C9—H9	0.9300
N3—C6	1.434 (8)	C10—C11	1.394 (8)
N3—H3	0.8600	C10—H10	0.9300
C1—C5	1.382 (8)	C11—H11	0.9300
C1—H1	0.9300	C12—Cl4	1.670 (15)
C2—C3	1.389 (11)	C12—Cl5	1.713 (17)
C2—H2	0.9300	C12—Cl3	1.746 (13)
C3—C4	1.342 (10)	C12—H12	0.9800
C3—H3A	0.9300		
			
N1—Hg1—Cl2	103.82 (14)	C1—C5—C4	115.5 (7)
N1—Hg1—Cl1	102.31 (14)	N3—C6—C7	114.6 (5)
Cl2—Hg1—Cl1	142.96 (7)	N3—C6—H6A	108.6
N1—Hg1—N2^i^	99.05 (17)	C7—C6—H6A	108.6
Cl2—Hg1—N2^i^	101.20 (14)	N3—C6—H6B	108.6
Cl1—Hg1—N2^i^	100.11 (13)	C7—C6—H6B	108.6
C2—N1—C1	120.0 (6)	H6A—C6—H6B	107.6
C2—N1—Hg1	120.0 (5)	C8—C7—C11	117.5 (5)
C1—N1—Hg1	119.7 (4)	C8—C7—C6	121.0 (5)
C10—N2—C9	115.6 (5)	C11—C7—C6	121.5 (6)
C10—N2—Hg1^ii^	122.4 (4)	C9—C8—C7	119.9 (6)
C9—N2—Hg1^ii^	122.0 (4)	C9—C8—H8	120.0
C5—N3—C6	123.7 (5)	C7—C8—H8	120.0
C5—N3—H3	118.2	N2—C9—C8	124.5 (6)
C6—N3—H3	118.2	N2—C9—H9	117.7
N1—C1—C5	123.7 (6)	C8—C9—H9	117.7
N1—C1—H1	118.2	N2—C10—C11	124.3 (6)
C5—C1—H1	118.2	N2—C10—H10	117.8
N1—C2—C3	120.6 (7)	C11—C10—H10	117.8
N1—C2—H2	119.7	C7—C11—C10	118.1 (6)
C3—C2—H2	119.7	C7—C11—H11	120.9
C4—C3—C2	120.3 (7)	C10—C11—H11	120.9
C4—C3—H3A	119.8	Cl4—C12—Cl5	110.8 (10)
C2—C3—H3A	119.8	Cl4—C12—Cl3	109.4 (9)
C3—C4—C5	119.9 (7)	Cl5—C12—Cl3	113.3 (11)
C3—C4—H4	120.1	Cl4—C12—H12	107.7
C5—C4—H4	120.1	Cl5—C12—H12	107.7
N3—C5—C1	123.1 (6)	Cl3—C12—H12	107.7
N3—C5—C4	121.4 (6)		
			
C2—N1—C1—C5	0.3 (9)	N3—C6—C7—C8	150.4 (6)
Hg1—N1—C1—C5	174.1 (4)	N3—C6—C7—C11	−29.1 (9)
C1—N1—C2—C3	0.6 (9)	C11—C7—C8—C9	−2.3 (10)
Hg1—N1—C2—C3	−173.3 (5)	C6—C7—C8—C9	178.2 (6)
N1—C2—C3—C4	−0.8 (11)	C10—N2—C9—C8	−1.6 (10)
C2—C3—C4—C5	0.1 (11)	Hg1^ii^—N2—C9—C8	179.0 (5)
C6—N3—C5—C1	0.1 (9)	C7—C8—C9—N2	2.3 (11)
C6—N3—C5—C4	−179.6 (5)	C9—N2—C10—C11	1.1 (10)
N1—C1—C5—N3	179.5 (5)	Hg1^ii^—N2—C10—C11	−179.5 (5)
N1—C1—C5—C4	−0.8 (9)	C8—C7—C11—C10	1.9 (10)
C3—C4—C5—N3	−179.7 (6)	C6—C7—C11—C10	−178.7 (6)
C3—C4—C5—C1	0.6 (9)	N2—C10—C11—C7	−1.4 (11)
C5—N3—C6—C7	−70.9 (7)		

Symmetry codes: (i) −*x*+1/2, *y*+1/2, −*z*+1/2; (ii) −*x*+1/2, *y*−1/2, −*z*+1/2.

### Hydrogen-bond geometry (Å, º)

*Cg*1 is the centroid of the N1/C1–C5 ring.

**Table:**

*D*—H···*A*	*D*—H	H···*A*	*D*···*A*	*D*—H···*A*
N3—H3···Cl2^iii^	0.86	2.78	3.467 (5)	138
C8—H8···Cl1^iv^	0.93	2.80	3.654 (6)	153
C6—H6*B*···*Cg*1^iii^	0.97	2.71	3.465 (7)	135

Symmetry codes: (iii) −*x*+1/2, −*y*+1/2, −*z*; (iv) *x*−1/2, *y*−1/2, *z*.

## References

[bb1] Brandenburg, K. (2010). *DIAMOND*. Crystal Impact GbR, Bonn, Germany.

[bb2] Bruker (2014). *APEX2*, *SAINT* and *SADABS*. Bruker AXS Inc., Madison, Wisconsin, USA.

[bb3] Chifotides, H. T. & Dunbar, K. R. (2013). *Acc. Chem. Res.* **46**, 894–906. 10.1021/ar300251k 23477406

[bb4] Furukawa, S., Reboul, J., Diring, S., Sumida, K. & Kitagawa, S. (2014). *Chem. Soc. Rev.* **43**, 5700–5734. 10.1039/c4cs00106k 24811425

[bb5] Ju, H., Lee, E., Moon, S.-H., Lee, S. S. & Park, K.-M. (2014). *Bull. Korean Chem. Soc.* **35**, 3658–3660.

[bb6] Khlobystov, A. N., Brett, M. T., Blake, A. J., Champness, N. R., Gill, P. M. W., O’Neill, D. P., Teat, S. J., Wilson, C. & Schröder, M. (2003). *J. Am. Chem. Soc.* **125**, 6753–6761. 10.1021/ja029048y 12769586

[bb7] Lee, E., Ju, H., Moon, S.-H., Lee, S. S. & Park, K.-M. (2015). *Bull. Korean Chem. Soc.* **36**, 1532–1535.

[bb8] Lee, E., Ryu, H., Moon, S.-H. & Park, K.-M. (2013). *Bull. Korean Chem. Soc.* **34**, 3477–3480.

[bb9] Leong, W. L. & Vittal, J. J. (2011). *Chem. Rev.* **111**, 688–764. 10.1021/cr100160e 20804195

[bb10] Matter, H., Nazaré, M., Güssregen, S., Will, D. W., Schreuder, H., Bauer, A., Urmann, M., Ritter, K., Wagner, M. & Wehner, V. (2009). *Angew. Chem. Int. Ed.* **48**, 2911–2916. 10.1002/anie.200806219 19294721

[bb11] Moon, S.-H., Cho, S. & Park, K.-M. (2014). *Acta Cryst.* E**70**, 389–391. 10.1107/S1600536814022922 PMC4257320 25484754

[bb12] Moon, S.-H., Kang, Y. & Park, K.-M. (2015). *Acta Cryst.* E**71**, 1287–1289. 10.1107/S205698901501837X PMC4645078 26594493

[bb13] Moon, S.-H. & Park, K.-M. (2014). *Acta Cryst.* E**70**, m233. 10.1107/S1600536814011465 PMC4051042 24940213

[bb14] Robson, R. (2008). *Dalton Trans.* pp. 5113–5131. 10.1039/b805617j 18813362

[bb15] Sheldrick, G. M. (2008). *Acta Cryst.* A**64**, 112–122. 10.1107/S0108767307043930 18156677

[bb16] Sheldrick, G. M. (2015). *Acta Cryst.* C**71**, 3–8.

[bb17] Silva, P., Vilela, S. M. F., Tomé, J. P. C. & Almeida Paz, F. A. (2015). *Chem. Soc. Rev.* **44**, 6774–6803. 10.1039/c5cs00307e 26161830

[bb18] Uemura, K., Kumamoto, Y. & Kitagawa, S. (2008). *Chem. Eur. J.* **14**, 9565–9576. 10.1002/chem.200800806 18780391

[bb19] Zhang, Z.-Y., Deng, Z.-P., Huo, L.-H., Zhao, H. & Gao, S. (2013). *Inorg. Chem.* **52**, 5914–5923. 10.1021/ic400055t 23634904

